# Physical fitness in children with Marfan and Loeys-Dietz syndrome: associations between cardiovascular parameters, systemic manifestations, fatigue, and pain

**DOI:** 10.1007/s00431-024-05456-z

**Published:** 2024-03-11

**Authors:** Jessica Warnink-Kavelaars, Lisanne E. de Koning, Annelies E. van der Hulst, Annemieke I. Buizer, Nicole Poissonnier, Laura E. Wijninga, Leonie A. Menke, Laura Muiño Mosquera, Lies Rombaut, Raoul H. H. Engelbert

**Affiliations:** 1 Department of Rehabilitation Medicine, Amsterdam UMC location University of Amsterdam, Meibergdreef 9, PO box 22660, 1100 DD Amsterdam, The Netherlands; 2 Amsterdam Movement Sciences, Rehabilitation and Development, Amsterdam, The Netherlands; 3 Faculty of Health, Center of Expertise Urban Vitality, Amsterdam University of Applied Sciences, Tafelbergweg 51, Amsterdam, The Netherlands; 4 Department of Pediatric Cardiology, Emma Children’s Hospital, Amsterdam UMC location University of Amsterdam, Meibergdreef 9, Amsterdam, The Netherlands; 5 Department of Rehabilitation Medicine, Amsterdam UMC location Vrije Universiteit Amsterdam, De Boelelaan 1117, Amsterdam, The Netherlands; 6 Department of Pediatrics, Emma Children’s Hospital, Amsterdam UMC location University of Amsterdam, Meibergdreef 9, Amsterdam, The Netherlands; 7 Department of Pediatrics, Division of Pediatric Cardiology, Ghent University Hospital, Corneel Heymanslaan 10, Ghent, Belgium; 8 Center for Medical Genetics, Ghent University Hospital, Ghent University, Corneel Heymanslaan 10, Ghent, Belgium

**Keywords:** Cardiovascular disease, Marfan syndrome, Loeys-Dietz syndrome, Physical fitness, Fatigue, Child

## Abstract

Children with Marfan (MFS) and Loeys-Dietz syndrome (LDS) report limitations in physical activities, sports, school, leisure, and work participation in daily life. This observational, cross-sectional, multicenter study explores associations between physical fitness and cardiovascular parameters, systemic manifestations, fatigue, and pain in children with MFS and LDS. Forty-two participants, aged 6–18 years (mean (SD) 11.5(3.7)), diagnosed with MFS (*n* = 36) or LDS (*n* = 6), were enrolled. Physical fitness was evaluated using the Fitkids Treadmill Test’s time to exhaustion (TTE) outcome measure. Cardiovascular parameters (e.g., echocardiographic parameters, aortic surgery, cardiovascular medication) and systemic manifestations (systemic score of the revised Ghent criteria) were collected. Pain was obtained by visual analog scale. Fatigue was evaluated by PROMIS® Fatigue-10a-Pediatric-v2.0-short-form and PROMIS® Fatigue-10a-Parent-Proxy-v2.0-short-form. Multivariate linear regression analyses explored associations between physical fitness (dependent variable) and independent variables that emerged from the univariate linear regression analyses (criterion *p* < .05). The total group (MFS and LDS) and the MFS subgroup scored below norms on physical fitness TTE *Z*-score (mean (SD) −3.1 (2.9); −3.0 (3.0), respectively). Univariate analyses showed associations between TTE *Z*-score aortic surgery, fatigue, and pain (criterion *p* < .05). Multivariate analyses showed an association between physical fitness and pediatric self-reported fatigue that explained 48%; 49%, respectively, of TTE *Z*-score variance (*F* (1,18) = 18.6, *p* ≤ .001, *r*^2^ = .48; *F* (1,15) = 16,3, *p* = .01, *r*^2^ = .49, respectively).

*    Conclusions*: Physical fitness is low in children with MFS or LDS and associated with self-reported fatigue. Our findings emphasize the potential of standardized and tailored exercise programs to improve physical fitness and reduce fatigue, ultimately enhancing the physical activity and sports, school, leisure, and work participation of children with MFS and LDS.

**Table:** 

**What is Known:** *• Marfan and Loeys-Dietz syndrome are heritable connective tissue disorders and share cardiovascular and systemic manifestations.* *• Children with Marfan and Loeys-Dietz syndrome report increased levels of disability, fatigue and pain, as well as reduced levels of physical activity, overall health and health-related quality of life.*	
**What is New:** *• Physical fitness is low in children with Marfan and Loeys-Dietz syndrome and associated with self-reported fatigue.* *• Our findings emphasize the potential of standardized and tailored exercise programs to improve physical fitness and reduce fatigue, ultimately enhancing the physical activity and sports, school, leisure, and work participation of children with Marfan and Loeys-Dietz syndrome.*

**What is Known:**

*• Marfan and Loeys-Dietz syndrome are heritable connective tissue disorders and share cardiovascular and systemic manifestations.*

*• Children with Marfan and Loeys-Dietz syndrome report increased levels of disability, fatigue and pain, as well as reduced levels of physical activity, overall health and health-related quality of life.*

**What is New:**

*• Physical fitness is low in children with Marfan and Loeys-Dietz syndrome and associated with self-reported fatigue.*

*• Our findings emphasize the potential of standardized and tailored exercise programs to improve physical fitness and reduce fatigue, ultimately enhancing the physical activity and sports, school, leisure, and work participation of children with Marfan and Loeys-Dietz syndrome.*

## Introduction

Marfan syndrome (MFS) [1] and Loeys-Dietz syndrome (LDS) [2] are heritable connective tissue disorders (HCTD) and share cardiovascular (aortic root dilation, mitral valve prolapse) and systemic (musculoskeletal, ophthalmic, pulmonary, skin, facial) manifestations [3, 4]. It is of importance to explore how these various cardiovascular and systemic manifestations, alongside symptoms such as fatigue and pain, influence the physical fitness of children with MFS and LDS. Such insights can provide a better understanding of a child’s sedentary behavior [5], their capacity to engage in physical activities [6], and their overall participation in daily life, encompassing school, sports, leisure, and work. Currently there is a paucity of studies reporting on how cardiovascular (e.g., aortic surgery, cardiovascular medication, mitral valve insufficiency) and systemic manifestations (e.g., musculoskeletal problems) influence physical fitness in children with MFS and LDS.

A clinical intervention in children with MFS demonstrated that even a simple activity like walking 10,000 steps a day had the potential to slow down aortic root dilation [7]. Studies with transgenic mouse models of MFS have shown that moderate dynamic exercise can mitigate the progression of cardiovascular phenotypes associated with MFS [8]. Furthermore, daily mild aerobic exercise has been shown to halt the progression of aortic aneurysms while improving aortic wall elasticity and elastin fiber structural integrity [9]. Then, reviews on pediatric cardiac rehabilitation [10] and obesity in children [11] reported improved physical fitness following rehabilitation exercise programs. Moreover, the benefits of regular physical activity are well-established, particularly in reducing cardiovascular risks in both the general population and patients with cardiovascular, as well as other chronic diseases [12, 13].

Systemic manifestations like musculoskeletal problems as well as symptoms of fatigue and pain were reported by parents and children with MFS to negatively influence their physical activities [14–16]. Children with HCTD, including MFS and LDS, also documented reduced levels of physical activity and increased disability. These results were accompanied by reports of increased fatigue and pain, as well as a decline in overall health, health-related quality of life, and mental health [17, 18]. It is worth noting that the 2020 guidelines from the World Health Organization on physical activity and sedentary behavior emphasize the positive association between physical activity and physical fitness [5]. Our previous study has also indicated that physical activity in children with HCTD, including MFS and LDS, is positively associated with physical fitness [6].

Children with other HCTD such as the Ehlers-Danlos syndromes [19] reported that fatigue and pain negatively influenced their physical activities [20–22]. Similarly, children with juvenile idiopathic arthritis scored significantly lower on physical fitness in terms of lower muscle strength, muscular endurance, and aerobic and anaerobic capacity compared to their healthy peers [13]. Moreover, these children reported increased pain and fatigue, and a physical activity intervention improved their physical activitiy levels [23].

This observational cross-sectional, multicenter study aims to investigate associations between physical fitness and cardiovascular parameters, systemic manifestations, fatigue, and pain in children with MFS and LDS. By investigating these associations, we seek to identify the key variables that contribute to decline in physical fitness. The outcomes of this study have substantial implications for the tailored management and care of children with MFS and LDS. Understanding physical fitness and its contributors can guide the development of standardized and tailored interventions to enhance physical fitness; physical activities; and sport, school, leisure, and work participation in the daily life of children with MFS and LDS.

## Methods

### Study design

The study design is an observational cross-sectional, multicenter study.

### Participants

Participants, aged 6–18 years, with MFS and LDS, were included in this study. Exclusion criteria were comorbid prominent chronic diseases affecting physical fitness, cognitive impairment (IQ < 80), wheelchair dependency, medical or psychiatric disorders that may affect the measurements in this study, a diagnosis of neonatal MFS, and a *Z*-score of aortic diameter of the sinus of Valsalva (SoV) > 5. Children were recruited at the Amsterdam University Medical Centers, Expert Center for Marfan syndrome and related disorders in the Netherlands, and by the Center for Medical Genetics of the Ghent University Hospital in Belgium.

### Procedures

The study project was approved by the Medical Ethics Review Committee of the Amsterdam UMC (2019_121) and the Ethical Committee of Ghent University Hospital (EC2019/1958). Participants and parents were invited by letter. Signing of the informed consent was performed by parents (participant < 12 years), or by parents and participants (participant between 12 and 16 years), or by the participant themselves (participants ≥ 16 years).

The Fitkids Treadmill Test (FTT), physical examination, echocardiography, and questionnaires (sociodemographic data; PROMIS® Fatigue-10a-Pediatric-v2.0-short-form; PROMIS® Fatigue-10a-Parent-Proxy-v2.0-short-form; VAS Pain) were conducted between March 2020 and September 2021. Pediatric cardiologists performed and evaluated the echocardiographic records and gave approval for performing the FFT. Pediatric physiotherapists conducted the FTT and pediatric rehabilitation physicians, pediatric physiotherapists, and pediatricians performed the physical examination. A collective Dutch-Belgium training program and multiple briefings to reduce bias between the centers were performed.

Between January 2023 and April 2023 two pediatric cardiologists re-evaluated the echocardiographic records of all participants, and two researchers and a pediatric rehabilitation physician re-evaluated the systemic score of the revised Ghent criteria of participants with MFS.

### Participant characteristics

Data on age (years and months), gender (male/female), height, and weight were obtained during history taking and physical examination. BMI (Body Mass Index) *Z*-scores were calculated [24].The sociodemographic data were collected by a custom-made questionnaire completed by the parents.

### Physical fitness

Physical fitness was evaluated using the Fitkids Treadmill Test’s time to exhaustion (TTE) outcome measure [25]. TTE is defined as the point at which the participant can no longer keep up with the incremental speed of the treadmill, despite strong verbal standardized encouragement. FTT shows good validity and reliability in children with chronic diseases [26].

### Cardiovascular parameters

Echocardiographic records of the heart and aorta were used to obtain cardiovascular parameters [27], e.g., aortic diameters (mm) (annulus, sinus of Valsalva (SoV), ascending aorta), mitral valve prolapse (yes/no), degree of mitral valve insufficiency (MI) (no/mild/moderate/moderate-severe/severe) [28], ejection fraction (%), left ventricular internal diameter (mm) in diastole, and aorta insufficiency (AI). To calculate the *Z*-scores of the SoV and LVIDd, the Detroit nomogram was used [29]. To calculate the *Z*-score of the ascending aorta, the Halifax nomogram was used [30]. Furthermore, heart rate, systolic and diastolic blood pressure, cardiovascular medication (CVM), aortic surgery (yes/no), date of surgery, and type of surgery were collected.

### Systemic manifestations

The presence of systemic manifestations in children with MFS was obtained during physical examination using the systemic score of the revised Ghent criteria. The patient’s medical records were examined for a history of previous pneumothorax, dural ectasia, and protrusio acetabuli. The systemic score assigns points based on clinical characteristics associated with MFS including wrist and/or thumb sign, pectus deformity, hindfoot and/or flat foot deformity, pneumothorax, dural ectasia, protrusio acetabuli, reduced upper segment/lower segment and increased arm/height, scoliosis or thoracolumbar kyphosis, reduced elbow extension, facial features, skin striae, myopia, and mitral valve prolapse [1]. The systemic score of the revised Ghent criteria provides an overall score with a maximum score of 20. A score of 7 or higher is considered abnormal*.* A systemic score subgroup of skeletal manifestations including hindfoot deformity, protrusio acetabuli, and scoliosis or thoracolumbar kyphosis was created to investigate the association between physical fitness and the occurrence of one or more of these skeletal systemic manifestations.

### Fatigue

The Patient Reported Outcomes Measurement Information System Fatigue 10a – Pediatric v2.0 Short (PROMIS® F-PS-SF) assesses self-reported fatigue in children between 8 and 18 years. The Patient Reported Outcomes Measurement Information System Fatigue 10a – Parent Proxy v2.0 Short form (PROMIS® F-PP-SF) assesses parent-reported fatigue in children 5–18 years. Both questionnaires contain 10 fatigue statements focusing on the intensity of fatigue and its impact on initiating and completing tasks, school, and social activities as experienced during the past 7 days.

The questionnaire demonstrates satisfactory psychometric properties and was found to discriminate well between the severity of disease [31, 32].

### Pain

VAS pain assesses subjective pain over the last week. The intensity of pain is scored on a 0–100 scale with 0 referring to “no pain” and 100 to “very severe pain.” Children between 8 and 18 years old performed a self-report. Children under 8 years were proxy-reported. The validity and reliability is good for VAS pain as demonstrated in children with chronic diseases [33].

### Statistical analyses

Online survey data were exported from the Castor database to the Statistical Package for Social Science (SPSS) version 26.0 for Windows for all statistical analyses. Data were analyzed for the total group of participants with MFS and LDS (*n* = 42) and for the subgroup participants with MFS (*n* = 36) separately. The group size of children with LDS (*n* = 6) was small, and these analyses were for explorative interpretation only. Data were checked for errors, missing values, and outliers. Missing data and outliers were excluded from the analyses. Physical fitness, participant characteristics, cardiovascular parameters, systemic manifestations, fatigue, and pain were analyzed by descriptive analyses. Normality of distributions was visually inspected by normality plots and tested by Shapiro–Wilk tests. Participants who underwent aortic surgery (*n* = 4) were excluded from the analyses of the size of the aortic SoV and the aortic ascendens.

Univariate linear regression analyses were used to find associations of physical fitness: TTE *Z*-score (dependent variable) with independent variables: participant characteristics, systemic manifestations (Table 1), cardiovascular parameters (Table 2), fatigue and pain (Table 3). Thereafter, a multiple regression (backward elimination; probability of *F* for entry = 0.05 and for removal = 0.10) was performed to analyze associations between physical fitness and the significant independent variables found in the univariate linear regression analyses. The value *r*^2^ represents how well the regression model explains observed data. To determine multicollinearity, a correlation matrix was performed between the significant tested independent variables on the univariate linear regression analyses [34].

**Table 1:** Participant characteristics and systemic manifestations

	Total group (*n* = 42)	MFS (*n* = 36)	LDS (*n* = 6)
Gender, male *n* (%)	19 (45.2)	16 (44.4)	3 (50)
Age (years), mean (SD)	11.5 (3.7)	11.2 (3.8)	13.1 (3.6)
BMI Z-score, mean (SD)	−1.0 (1.3)	−1.0 (1.3)	−0.8 (1.4)
Systemic score of the revised Ghent criteria, mean (SD)	n.a	5.8 (3.2)^a^	n.a
Systemic score > 7, *n* (%)	n.a	13 (36%)^a^	n.a
Skeletal manifestations^b^, *n* (%)	n.a	24 (66%)^a^	n.a
Lens luxation, *n* (%)	12 (29.3)	12 (34.3)	0 (0)
Genetic confirmation, *n* (%)	40 (95.2)	34 (94.4)^c^	6 (100)

*BMI* body mass index, *LDS* Loeys-Dietz syndrome, *MFS* Marfan syndrome, *n.a.* not applicable, *SD* standard deviation

^a^Missing 1

^b^Occurrence of one or more skeletal manifestations including hindfoot deformity, protrusio acetabuli and scoliosis or thoracolumbar kyphosis

^c^Two children had a clinical MFS confirmation

**Table 2:** Cardiovascular parameters

	Total group (*n* = 42)	MFS (*n* = 36)	LDS (*n* = 6)
General cardiovascular characteristics			
Heartrate (beats per minute), mean (SD)	72.1 (2.6)	72.5 (3.0)	69.7 (3.8)
Systolic blood pressure (mmHG), mean (SD)	102.0 (1.9)	101.8 (2.0)	104.3 (5.8)
Diastolic blood pressure (mmHG), mean (SD)	65.8 (1.3)	65.7 (1.5)	66.8 (1.1)
Use of CVM, *n* (%)	19 (45.2)	15 (41.7)	4 (66.7)
Aortic surgery, *n* (%)	4 (9.5)	2 (5.6)	2 (33.3)
Echocardiographic characteristics			
Aortic SoV diameter (mm), mean (SD)^a^	30.8 (5.1)	31.0 (5.1)	29.2 (5.7)
Aortic SoV *Z*-score, mean (SD)^a^	2.3 (1.2)	2.3 (1.2)	2.2 (1.3)
Aorta ascendens diameter (mm), mean (SD)^a^	23.1 (3.2)	23.3 (3.2)	21.3 (3.3)
Aorta ascendens *Z*-score, mean (SD)^a^	1.3 (1.3)	1.4 (1.3)	0.8 (0.8)
Aortic insufficiency, *n* (%)	5 (13.2)	3 (9.1)	2 (40.0)
Mitral valve prolapse, *n* (%)	18 (42.9)	16 (44.4)	2 (33.3)
Mitral valve insufficiency, *n* (%)	19 (46.3)	17 (47.2)	2 (33.3)
Mild, *n* (%)	11 (26.8)	10 (27.8)	1 (16.7)
Moderate, *n* (%)	8 (19.5)	7 (19.4)	1 (16.7)
Ejection fraction (%), mean (SD)	54.8 (6.2)	55.0 (6.5)	53.7 (4.9)
LVIDd (mm), mean (SD)	46.4 (5.9)	46.8 (6.1)	43.4 (4.4)
LVIDd *Z*-score, mean (SD)	0.4 (1.0)	0.5 (1.1)	0.2 (0.5)

*CVM* cardiovascular medication, *LDS* Loeys-Dietz syndrome, *LVIDd* left ventricular internal diameter in diastole, *MFS* Marfan syndrome, *SD* standard deviation, *SoV* sinus of Valsalva

^a^Of the participants who underwent aortic surgery (*n* = 4, MFS *n* = 2, LDS *n* = 2), the echocardiographic parameters of the aorta were not included, and therefore, the aortic SoV and the aortic ascendens diameters (mm) were not calculated

**Table 3:** Fatigue and pain

	Total group (*n* = 42)	MFS (*n* = 36)	LDS (*n* = 6)
PROMIS® F-PP-SF			
* Z*-score, mean (SD)	0.3 (1.2)^a^	0.4 (1.2)^b^	0.7 (0.5)^c^
PROMIS® F-SP-SF			
* Z*-score, mean (SD)	1.0 (0.9)^d^	1.0 (0.9)^e^	0.9 (0.8)^f^
VAS Pain			
* Z*-scores, mean (SD)	0.8 (0.9)^a^	0.8 (0.9)^b^	0.9 (1.2)^c^

*LDS* Loeys-Dietz syndrome, *MFS* Marfan syndrome, *PROMIS®* *F-PP-SF* Patient Reported Outcomes Measurement Information System Fatigue Parent Proxy Short Form, *PROMIS®* *F-SP-SF* Patient Reported Outcomes Measurement Information System Fatigue Self-reported Pediatric Short Form, *SD* standard deviation, *VAS* visual analog scale

^a^Missing 9

^b^Missing 6

^c^Missing 3

^d^Missing 8

^e^Missing 6

^f^Missing 2

## Results

### Participant characteristics and systemic manifestations

A cohort of 42 participants, (45% male), aged 6–18 years (mean (SD) 11.5 (3.7)), diagnosed with MFS (*n* = 36; genetically confirmed MFS *n* = 34 (*FBN1* 34 participants), clinically confirmed MFS *n* = 2) and LDS (*n* = 6; genetically confirmed LDS, *n* = 6 (*TGFBR1* 2 participants, *TGFBR2* 3 participants, *TGFB3* 1 participant)), were enrolled. Participants with MFS scored on the systemic subscores of the revised Ghent criteria *n* (%): wrist and/or thumb sign 27 (77%), pectus deformity 19 (54%); hindfoot and/or flat feet deformity 21 (60%); pneumothorax 1 (3%); dural ectasia 1 (3%), protrusio acetabuli 1 (3%), reduced upper segment/lower segment and increased arm/height 5 (14%); scoliosis or thoracolumbar kyphosis 12 (34%); reduced elbow extension 3 (9%); facial features 19 (54%); skin striae 10 (29%), myopia 10 (29%); mitral valve prolapse 16 (44%). The total systemic score of the revised Ghent criteria was mean (SD) 5.8 (3.2); 13 (36%) participants achieved a systemic score of > 7 (Table 1). Of all participants 24 (66%) showed one or more skeletal manifestations including hindfoot deformity, protrusio acetabuli, and scoliosis/thoracolumbar kyphosis (Table 1).

### Physical fitness

TTE Z-scores for the total group and MFS subgroup were mean (SD) −3.1 (2.9) and −3.0 (3.0), respectively.

### Cardiovascular parameters

#### Total group

Almost half of the participants (45.2%) used cardiovascular medication (MFS *n* = 15, LDS *n* = 4). Atenolol was used by nine and Losartan by ten children. Two participants with MFS underwent a Personalized External Aortic Root Support (PEARS) procedure and two participants with LDS underwent a valve sparing aortic root replacement (David procedure); two participants underwent their surgery less than 1 year before conducting the FTT.

Participants showed an aortic SoV *Z*-score of mean (SD) 2.3 (1.2); 52% had a SoV *Z*-score ≥ 2, and 21% had a SoV Z-score ≥ 3. The aortic ascendens *Z*-score was mean (SD) 1.3 (1.3). Mitral valve insufficiency was recorded in 46%, which was mild in 26.8% and moderate in 19.5% (Table 2).

#### MFS subgroup

The aortic SoV *Z*-score was mean (SD) 2.3 (1.2); 50% showed a SoV *Z*-score ≥ 2, and 18% showed a SoV *Z*-score ≥ 3. The aortic ascendens *Z*-score was mean (SD) 1.4 (1.3). Mitral valve insufficiency was recorded in 47.2% which was mild in 27.8% and moderate in 19.4% (Table 2).

### Fatigue and pain

#### Total group

Pediatric self-reported fatigue (PROMIS® F-SP-SF Z-score) was mean (SD) 1.0 (0.9). Parent proxy reported fatigue (PROMIS® F-PP-SF *Z*-score) was mean (SD) 0.3 (1.2). VAS pain *Z*-score over the last 7 days was mean (SD) 0.8 (0.9) (Table 3).

#### MFS subgroup

Pediatric self-reported fatigue PROMIS® F-SP-SF *Z*-score was mean (SD) 1.0 (0.9). Parent proxy reported fatigue PROMIS® F-PP-SF Z-score was mean (SD) 0.4 (1.2). VAS pain *Z*-score over the last 7 days was mean (SD) 0.8 (0.9) (Table 3).

### Univariate linear regression

#### Total group

Pediatric self-reported fatigue (PROMIS® F-SP-SF *Z*-score), parent proxy fatigue (PROMIS® F-PP-SF *Z*-score), pain (VAS pain *Z*-score), and aortic surgery were associated with physical fitness (TTE *Z*-score) (criterion, *p* < .05). Pediatric self-reported fatigue explained 48.2% of the variance in scores (*F* (1, 21) = 19.567, *p* < .001, *r*^2^ = .482). Parent proxy fatigue explained 14.6% of the variance in scores (*F* (1, 25) = 4.287, *p* = .049, *r*^2^ = .146). Pain explained 25.0% of the variances in scores (*F* (1, 28) = 9.340, *p* = .005, *r*^2^ = .250). Previous aortic surgery explained 21.7% of the variance in scores (*F* (1, 35) = 9.673, *p* = .004, *r*^2^ = .217). Analyses indicate that higher levels of fatigue, pain, and previous aortic surgery are associated with lower physical fitness. Participant characteristics (Table 1) and cardiovascular parameters (Table 2) did not associate with physical fitness (criterion, *p* < .05).

#### MFS subgroup

Pediatric reported fatigue (PROMIS® F-SP-SF *Z*-score), pain (VAS pain *Z*-score), and aortic surgery associated with physical fitness (TTE *Z*-score) (criterion, *p* < .05). Pediatric reported fatigue explained 49.3% of the variance in scores (*F* (1, 18) = 17.508, *p* < .001, *r*^2^ = .493). Pain explained 52.7% of the variances in scores (*F* (1, 25) = 9.622, *p* = .005, *r*^2^ = .527). Previous aortic surgery explained 20.6% of the variance in scores (*F* (1, 30) = 7.776, *p* = .009, *r*^2^ = .206). Analyses indicate that higher levels of fatigue, pain, and previous aortic surgery are associated with lower physical fitness. Participant characteristics (Table 1), systemic manifestations (all separate systemic subscores of the revised Ghent criteria; total systemic score of the revised Ghent criteria, total systemic score > 7 and occurrence of one or more skeletal manifestations including hindfoot deformity, protrusio acetabuli, and scoliosis/thoracolumbar kyphosis), and cardiovascular parameters (Table 2) were not associated with physical fitness (criterion, *p* < .05).

### Multivariate regression analyses

#### Total group

Multivariate backward regression analyses indicated that 48% of the variance in TTE was explained by pediatric self-reported fatigue (PROMIS® F-SP-SF *Z*-score) (*F* (1,18) = 18.6, *p* ≤ .001, *r*^2^ = .48). Analyses indicate that higher levels of pediatric self-reported fatigue are associated with lower physical fitness. Parent proxy fatigue (PROMIS® F-PP-SF *Z*-score), pain (VAS pain *Z*-score), and previous aortic surgery were not significantly associated with physical fitness (criterion, *p* < .05).

There was a small correlation between pediatric self-reported fatigue (PROMIS® F-SP-SF Z-score) and pain (VAS pain *Z*-score) (*r* = .458, *p* < .021) and no significant correlations between aortic surgery (*r* = .299, *p* < .138) (criterion, *p* < .05). This indicates no multicollinearity.

#### MFS subgroup

Multivariate backward regression analyses indicated that 49% of the variance in TTE was explained by pediatric self-reported fatigue (PROMIS® F-SP-SF *Z*-score) (*F* (1,15) = 16,3, *p* = .01*, r*^2^ = .49). VAS pain *Z*-score and aortic surgery were not significantly associated with physical fitness (criterion, *p* < .05).

There were no significant correlations between pediatric self- reported fatigue and pain (VAS pain *Z*-score) and aortic surgery (*r* = .409, *p* < .066; *r* = ,316, *p* < .151, respectively). This indicates no multicollinearity.

## Discussion

Our study demonstrated low physical fitness among children with MFS [1] and LDS [2] compared to the norm. In our multivariate analyses, a moderate association emerged between physical fitness and self-reported fatigue, as reflected by a robust *r*^2^ value of 0.48. This indicates that heightened self-reported fatigue levels are associated with low physical fitness. This is in line with a previous study that suggested that low physical fitness contributed to elevated fatigue levels in various chronic childhood conditions [13]. Our findings emphasize the potential of standardized and tailored exercise programs to improve physical fitness and reduce fatigue, ultimately enhancing the physical activity and participation of children with MFS and LDS. It is essential that medical professionals (e.g., physiotherapists, cardiologists, pediatricians, rehabilitation physicians) actively discuss and encourage both children and their parents to participate in sports and exercise programs.

Our univariate analyses demonstrated associations between physical fitness and aortic surgery and pain. These associations did not reach statistical significance in the multivariate analyses. Reviews reported improvement of physical fitness of children with aortic surgery [10] and pediatric obesity [11] following physical exercise programs. Children with MFS and LDS reported that pain substantially influenced their physical activity levels and participation in daily life [14–16] as well as their health related quality of life [17, 18]. Therefore, medical professionals may also consider factors, like aortic surgery, pain, and other factors not covered in our study (e.g., health-related quality of life), in addition to fatigue when treating an individual patient with low physical fitness.

Cardiovascular parameters and systemic manifestations [3, 4] were not significantly associated with physical fitness in our multivariate analyses. Studies on physical activity in children with MFS showed the potential to slow down aortic root dilation [7]. Transgenic mouse models of MFS reported that moderate dynamic exercise mitigated the progression of cardiovascular phenotypes associated with MFS [8, 9]. Regular physical activity reduced cardiovascular risks in the general population and in patients with cardiovascular diseases and other chronic childhood conditions [12]. Furthermore, children with MFS reported about the adverse impact of systemic manifestations, such as musculoskeletal problems, on their physical activities [14–16]. Yet, our study findings revealed no significant associations between FTT and the most likely cardiovascular parameters as MI, AI, aortic SoV *Z*-score, aortic surgery, heartrate, blood pressure, and the systemic score of the revised Ghent criteria which includes musculoskeletal manifestations. It is noteworthy that the occurrence of patients with MI, AI, and aortic surgery, as well as the aortic SoV Z-score and the systemic score of the revised Ghent criteria, were relatively low in our study. Then, heartrate and blood pressure showed low variability. These low distributions may complicate drawing conclusions due to data homogeneity. Furthermore, while the systemic score of the revised Ghent criteria encompasses musculoskeletal manifestations, in future studies focusing on physical fitness, the exploration of a comprehensive musculoskeletal subset of systemic manifestations would provide significant value.

Therefore, a larger sample including more severely affected patients would enhance the robustness of our results.

Our study has several strengths. A wide range of potential contributors to physical fitness by encompassing cardiovascular, systemic, fatigue, and pain variables were addressed. The lack of multicollinearity in our study increased our confidence in the accuracy of the association between physical fitness and fatigue. Furthermore, established measures for assessing physical fitness, cardiovascular parameters, systemic manifestations, fatigue, and pain, along with normative scores for children, were used. These validated tools boost result dependability and comparability, enhancing the overall credibility of our findings.

Study limitations should be acknowledged. The sample size for the LDS group was relatively small, and these analyses were for explorative interpretation only. Our study included a limited number of patients with severe cardiovascular and systemic manifestations. Limited variability in the distribution of data could create challenges in reaching conclusions. Increasing the sample size to include a greater number of patients with more severe cardiovascular and systemic manifestations would strengthen the reliability of our findings [34].

In conclusion, physical fitness is low in children with MFS or LDS and associated with self-reported fatigue. Our findings emphasize the potential of standardized and tailored exercise programs to improve physical fitness and reduce fatigue, ultimately enhancing the physical activity and sports, school, leisure, and work participation of children with MFS and LDS.

## Data Availability

The data that support the findings of this study are available on request from the corresponding author, JW. The data are not publicly available due to restrictions e.g. their containing information that could compromise the privacy of research participants.

## Abbreviations

AI: Aorta insufficiency
BMI: Body mass index
CVM: Cardiovascular medication
FTT: Fitkids Treadmill Test
HCTD: Heritable connective tissue disorders
LDS: Loeys-Dietz syndrome
MFS: Marfan syndrome
MI: Mitral valve insufficiency
PROMIS: Patient-Reported Outcomes Measurement Information Systems
SoV: Sinus of Valsalva
TTE: Time to exhaustion
VAS: Visual analog scale
